# Supplementary material for: Modelling Skylarks (Alauda arvensis) to Predict Impacts of Changes in Land Management and Policy: Development and Testing of an Agent-Based Model
Source: PLoS One. 2013 Jun 6;8(6):e65803. doi: 10.1371/journal.pone.0065803 (PMC3675089; doi:10.1371/journal.pone.0065803)
Supplement: Supporting Information S4 — The skylark ODdox as a zipped archive. (ZIP) [file pone.0065803.s004.zip › Skylark_ODdox/class_map_error_msg.html]

ALMaSS Skylark ODdox: MapErrorMsg Class Reference


ALMaSS Skylark ODdox
 2.0


- Main Page
- Related Pages
- Classes
- Files

- Class List
- Class Index
- Class Hierarchy
- Class Members

Public Member Functions |
Private Attributes

MapErrorMsg Class Reference

`#include <maperrormsg.h>`

List of all members.

|  |  |
| --- | --- |
| Public Member Functions | |
|  | MapErrorMsg (const char \*a\_warnfile) |
| void | SetWarnLevel (MapErrorState a\_level) |
| void | Warn (MapErrorState a\_level, const char \*a\_msg1, const char \*a\_msg2) |
| void | Warn (const char \*a\_msg1, const char \*a\_msg2) |
| void | Warn (const char \*a\_msg1, double a\_num) |
| void | WarnAddInfo (MapErrorState a\_level, const char \*a\_add1, const char \*a\_add2) |

|  |  |
| --- | --- |
| Private Attributes | |
| MapErrorState | m\_level |
| const char \* | m\_warnfile |

---

## Constructor & Destructor Documentation

|  |  |  |  |  |  |
| --- | --- | --- | --- | --- | --- |
| MapErrorMsg::MapErrorMsg | ( | const char \* | *a\_warnfile* | ) |  |

References m\_warnfile.

{

m\_warnfile = a\_warnfile;

}

---

## Member Function Documentation

|  |  |  |  |  |  |
| --- | --- | --- | --- | --- | --- |
| void MapErrorMsg::SetWarnLevel | ( | MapErrorState | *a\_level* | ) |  |

References m\_level, Warn(), WARN\_ALL, and WARN\_BUG.

Referenced by Landscape::Landscape().

{

if ( a\_level > WARN\_BUG && a\_level <= WARN\_ALL )

m\_level = a\_level;

else {

m\_level = WARN\_ALL;

Warn( WARN\_BUG, "MapErrorMsg::SetWarnLevel(): Illegal error level!", "");

exit(1);

}

}

|  |  |  |  |
| --- | --- | --- | --- |
| void MapErrorMsg::Warn | ( | MapErrorState | *a\_level*, |
|  |  | const char \* | *a\_msg1*, |
|  |  | const char \* | *a\_msg2* |
|  | ) |  |  |

References m\_warnfile.

Referenced by Landscape::AddBeetleBanks(), LE\_TypeClass::BackTranslateEleTypes(), LE\_TypeClass::BackTranslateVegTypes(), Landscape::BorderAdd(), Landscape::BorderNeed(), Skylark\_Population\_Manager::BreedingPairsOutput(), Skylark\_Population\_Manager::BreedingSuccessProbeOutput(), Landscape::ChangeMapMapping(), Farm::CheckRotationManagementLoop(), FarmManager::ConnectFarm(), FarmManager::CreateFarms(), CropRotation::CropRotation(), Calendar::DayLength(), RodenticideManager::DoPlaceBait(), Landscape::DumpMap(), Landscape::DumpMapGraphics(), SkTerritories::DumpMapGraphics(), Landscape::DumpMapInfoByArea(), Configurator::DumpSymbols(), Landscape::DumpTreatCounters(), Landscape::EventDump(), Landscape::EventtypeToString(), Configurator::ExtractString(), probe\_data::FileAppendOutput(), CropData::FindCropNum(), Skylark\_Population\_Manager::FledgelingProbeOutput(), Landscape::ForceArea(), RasterMap::Get(), CropRotation::GetFirstCrop(), CropRotation::GetNextCrop(), Landscape::IncTreatCounter(), MovementMap::Init(), Farm::InitiateManagement(), UserDefinedFarm::InvIntPartition(), Landscape::Landscape(), Farm::LeSwitch(), Farm::Management(), Landscape::NewElement(), probe\_data::OpenFile(), probe\_data::OpenForAppendToFile(), Skylark\_Population\_Manager::OpenTheBreedingPairsProbe(), Skylark\_Population\_Manager::OpenTheBreedingSuccessProbe(), Population\_Manager::OpenTheCIPEGridOutputProbe(), Population\_Manager::OpenTheMonthlyRipleysOutputProbe(), Population\_Manager::OpenTheReallyBigProbe(), Population\_Manager::OpenTheRipleysOutputProbe(), Landscape::OrchardBorderAdd(), Configurator::ParseCfgLine(), Pesticide::Pesticide(), Landscape::PolysDump(), Landscape::PolysValidate(), Landscape::PolytypeToString(), SkTerritories::PreFillQualGrid(), RasterMap::RasterMap(), VegElement::ReadBugPercentageFile(), Landscape::ReadPolys(), Farm::ReadRotation(), Configurator::ReadSymbols(), Farm::RemoveField(), Pesticide::SavePPM(), Configurator::SetCfgBool(), Configurator::SetCfgFloat(), Configurator::SetCfgGatekeeper(), Configurator::SetCfgInt(), Configurator::SetCfgStr(), CropData::SetVegNum(), SetWarnLevel(), Skylark\_Female::st\_Finding\_Territory(), Skylark\_Female::st\_Laying(), Skylark\_Female::st\_MakingNest(), Farm::TranslateCropCodes(), LE\_TypeClass::TranslateEleTypes(), LE\_TypeClass::TranslateVegTypes(), Landscape::UnsprayedMarginAdd(), UserDefinedFarm::UserDefinedFarm(), Landscape::VegDump(), CropData::VegTypeToCurveNum(), Landscape::VegtypeToString(), Landscape::Warn(), and Weather::Weather().

{

FILE \* EFile;

time\_t aclock;

tm\* newtime;

time( &aclock ); // Get time in seconds.

newtime=localtime( &aclock ); // Convert time to struct tm form.

// Print local time as a string

EFile=fopen(m\_warnfile, "a+" );

if ( !EFile ) {

fprintf(stderr, "MapErrorMsg::Warn(): Unable to open file"

" for error messages: %s\n", m\_warnfile );

exit(1);

}

fprintf( EFile, "%s Level %d \*\*\*\*\*\n%s %s\n",

asctime(newtime), a\_level, a\_msg1, a\_msg2 );

fflush( EFile );

fclose( EFile );

}

|  |  |  |  |
| --- | --- | --- | --- |
| void MapErrorMsg::Warn | ( | const char \* | *a\_msg1*, |
|  |  | const char \* | *a\_msg2* |
|  | ) |  |  |

References m\_warnfile.

{

FILE \* EFile;

time\_t aclock;

tm\* newtime;

time( &aclock ); // Get time in seconds.

newtime=localtime( &aclock ); // Convert time to struct tm form.

// Print local time as a string.

EFile=fopen(m\_warnfile, "a+" );

if ( !EFile ) {

fprintf(stderr, "MapErrorMsg::Warn(): Unable to open file"

" for error messages: %s\n", m\_warnfile );

exit(1);

}

fprintf( EFile, "%s \*\*\*\*\*\n%s %s\n",

asctime(newtime), a\_msg1, a\_msg2 );

fflush( EFile );

fclose( EFile );

}

|  |  |  |  |
| --- | --- | --- | --- |
| void MapErrorMsg::Warn | ( | const char \* | *a\_msg1*, |
|  |  | double | *a\_num* |
|  | ) |  |  |

References m\_warnfile.

{

FILE \* EFile;

time\_t aclock;

tm\* newtime;

time( &aclock ); // Get time in seconds.

newtime=localtime( &aclock ); // Convert time to struct tm form.

// Print local time as a string.

EFile=fopen(m\_warnfile, "a+" );

if ( !EFile ) {

fprintf(stderr, "MapErrorMsg::Warn(): Unable to open file"

" for error messages: %s\n", m\_warnfile );

exit(1);

}

fprintf( EFile, "%s \*\*\*\*\*\n%s %g\n",

asctime(newtime), a\_msg1, a\_num );

fflush( EFile );

fclose( EFile );

}

|  |  |  |  |
| --- | --- | --- | --- |
| void MapErrorMsg::WarnAddInfo | ( | MapErrorState | *a\_level*, |
|  |  | const char \* | *a\_add1*, |
|  |  | const char \* | *a\_add2* |
|  | ) |  |  |

References m\_level, and m\_warnfile.

Referenced by Configurator::ShowIdType().

{

FILE \* EFile;

if ( a\_level > m\_level )

return;

EFile=fopen(m\_warnfile, "a+" );

if (!EFile) {

fprintf(stderr, "MapErrorMsg::Warn(): Unable to open file"

" for error messages: %s\n", m\_warnfile );

exit(1);

}

fprintf( EFile, "%s%s", a\_add1, a\_add2 );

fflush( EFile );

fclose( EFile );

}

---

## Member Data Documentation

MapErrorState MapErrorMsg::m\_level

private

Referenced by SetWarnLevel(), and WarnAddInfo().

const char\* MapErrorMsg::m\_warnfile

private

Referenced by MapErrorMsg(), Warn(), and WarnAddInfo().

---

The documentation for this class was generated from the following files:

- maperrormsg.h
- maperrormsg.cpp


- MapErrorMsg
- Generated on Thu Jan 10 2013 13:15:36 for ALMaSS Skylark ODdox by
   1.8.1.1
